# Seagrass *Halodule wrightii* as a new habitat for the amphioxus *Branchiostoma californiense* (Cephalochordata, Branchiostomidae) in the southern Gulf of California, Mexico

**DOI:** 10.3897/zookeys.873.33901

**Published:** 2019-08-29

**Authors:** Lucía Campos-Dávila, Claudia J. Pérez-Estrada, Ricardo Rodríguez-Estrella, Enrique Morales-Bojórquez, Fernando G. Brun-Murillo, Eduardo F. Balart

**Affiliations:** 1 Centro de Investigaciones Biológicas del Noroeste, S.C., Av. Instituto Politécnico Nacional 195, Col. Playa Palo de Santa Rita Sur, La Paz Baja California Sur, México Centro de Investigaciones Biológicas del Noroeste La Paz Mexico; 2 Universidad de Cádiz, Departamento de Biología, Estructura y Dinámica de Ecosistemas Acuáticos, Puerto Real, Cádiz, Spain Universidad de Cádiz Puerto Real Spain

**Keywords:** Allometric growth pattern, amphioxi, isometric growth pattern, morphological variability, new habitat, sex size structure

## Abstract

The first record of the amphioxus *Branchiostoma californiense* on seagrass patches of *Halodule wrightii* in the Gulf of California is reported. Sixty individuals (19 males, 18 females, and 23 undifferentiated) were collected in May 2017 at Bahía Balandra, Gulf of California, from subtidal seagrass patches at a depth of 0.5 m at low tide. The length and weight ranged from 15.88–28.44 mm and from 0.01–0.11 g for females and 11.7–27.9 mm and 0.01–0.09 g for males, respectively. The minimum size of sexually mature individuals was 11.70 mm for males and 15.88 mm for females; 62% of the specimens were sexually mature. Analysis of the total length-weight relationship suggested an allometric growth pattern among females, males and undifferentiated individuals, whereas an analysis of the entire sample suggested an isometric growth pattern. Typical and additional morphological characters were used to identify the amphioxi. High morphological variability between individuals was found, suggesting the presence of several morphotypes. *Branchiostoma californiense* had been previously reported as exclusively associated with bare sandy areas, but our study shows that this species can also be found in seagrass patches, using them as breeding and feeding grounds. Thus, seagrass patches are evidenced as suitable habitats for amphioxus.

## Introduction

Amphioxi are Cephalochordata often used as a model for studying the phylogeny and evolution of vertebrates (Stokes and Holland 1998, Bertrand and Escriva 2011, Vergara et al. 2012). Thus, research on this group has mainly focused on morphology, genetics (Holland 2010), embryology (Desdevises et al. 2011), and evolution (Putnam et al. 2008). Amphioxi are marine organisms inhabiting shallow waters near the coast, such as estuaries, coastal lagoons, open coasts, and even river deltas, in temperate and tropical waters (Laudien et al. 2007, Meerhoff et al. 2016). They are benthic, obligate filter feeders that play a key role in the transfer of microbial and phytoplanktonic production to higher trophic levels, including fishes (Ruppert et al. 2000, Vargas and Dean 2010). *Branchiostoma californiense* Andrews, 1893, is the only species reported for the eastern Pacific coast in Central and North America (Vargas 1987, Poss and Boschung 1996, Vargas and Dean 2010). In Mexico, it is distributed from the northwestern coast of the Baja California Peninsula to the coast of Oaxaca, including the Gulf of California; some records from Central America have also been reported (Poss and Boschung 1996, Vargas and Dean 2010, Del Moral-Flores et al. 2016). Two recent studies describe the occurrence and taxonomy of *B. californiense* in Mexico based on preserved specimens. Del Moral-Flores et al. (2016) examined the taxonomic composition and distribution of cephalochordates (Cephalochordata: Amphioxiformes) in Mexico based on specimens from collections of various institutions. Galván-Villa et al. (2017) reported new records of *B. californiense* for the central Mexican Pacific and included a taxonomic description of the species based on five specimens. In this study, we aim to highlight seagrass as a novel and likely suitable habitat for *B. californiense* during breeding in the southern part of the Gulf of California, as well as to add to the knowledge of its taxonomy and biology.

## Study area

**Figure 1.**
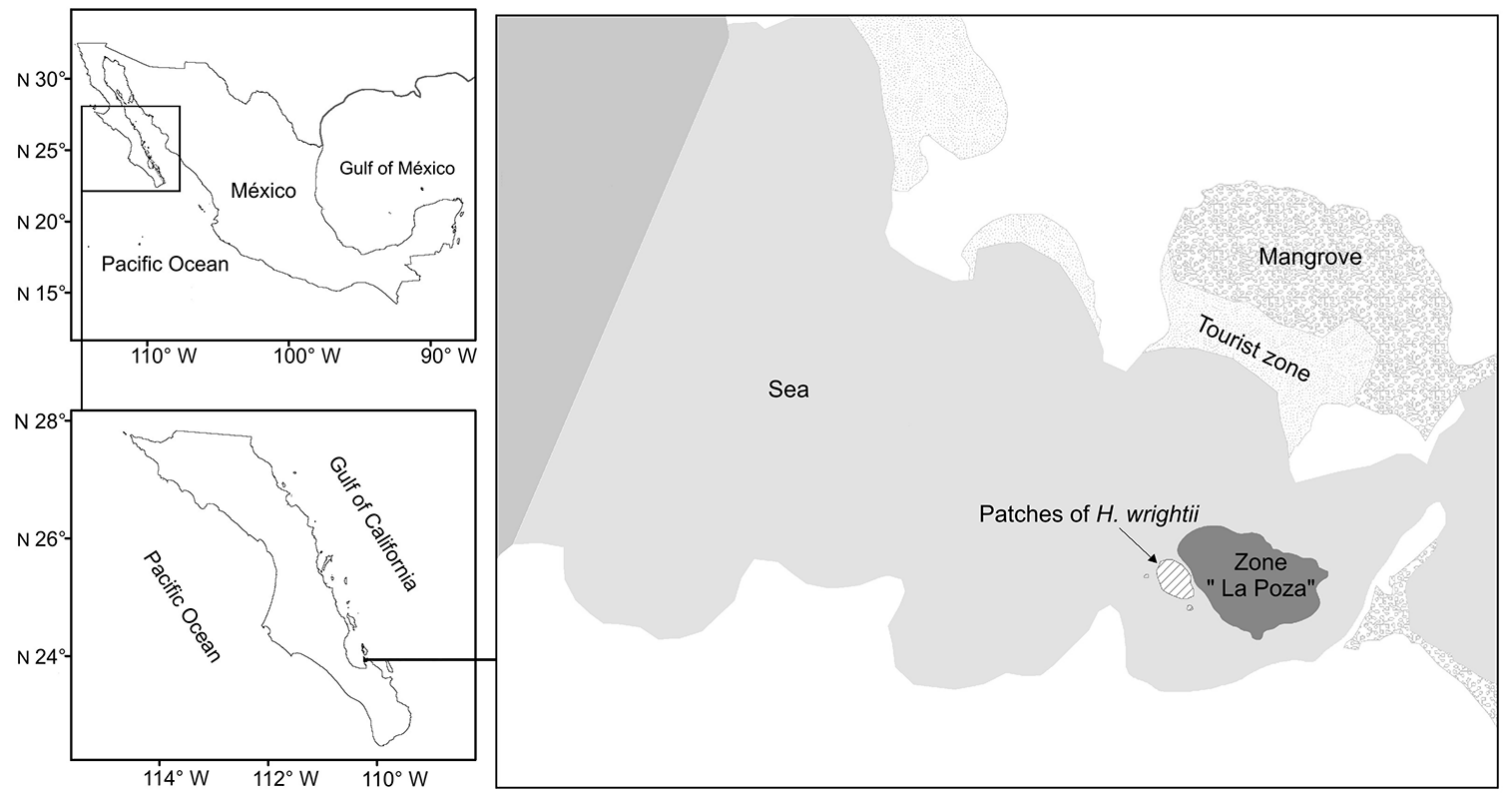
Amphioxus study area, Bahía Balandra southern Baja California Peninsula.

Bahía Balandra (24°18'54.8"N, 110°19'39.3"W) is a natural protected area located in Bahía de La Paz, Mexico, in the southern part of the Gulf of California (Fig. 1). Bahía Balandra is a coastal lagoon covering an area of 2512.5 ha where coastal vegetation is dominated by three mangrove species (*Rhizophora mangle*, *Laguncularia racemosa* and *Avicennia germinans*). Rocky reefs (Félix-Pico 2009) and patches of the seagrass *Halodule wrightii* also occur in the bay. The beach is gently sloping, shallow, and usually waveless; the tidal range is 1.0–1.5 m with a semidiurnal pattern. The climate is arid, and rainfall is limited to sporadic winter storms or summer hurricanes. Sediment in the bay consists of fine to coarse sand of mixed siliciclastic-calcareous composition (López 2013).

## Materials and methods

### Habitat

In 2017, a few individuals of the amphioxus *B. californiense* were observed during the course of surveys to monitor the metabolism of seagrass communities. In May 2017, 60 amphioxus specimens were collected in the area called La Poza in Bahía Balandra from a monospecific seagrass patch measuring approximately 24 m^2^. Amphioxus density (individuals/m^2^) was estimated by burying a 4.5 L bucket in the sand and dragging it horizontally in the seagrass patch ten times; a total area of 0.75 m^2^ (0.50 m x 0.15 m x 10 m) was thus sampled. The specimens were placed in plastic bottles containing a bed of sand and seawater for transportation to the laboratory. Additional samplings were carried out to search for amphioxi in other areas of Bahía Balandra: i) in four seagrass patches near the sampled patch, ii) in bare sand areas 10 m away from the sampled patch and iii) in zones farther away (50–450 m) from the sampled patch. Environmental parameters were also recorded within the *H. wrightii* canopy. Water temperature (°C) was recorded during 24 hours using a HOBO data logger (UA-002–64). pH and salinity were measured at the beginning and end of the sampling with a multi-parameter YSI sonde. Chlorophyll concentration in water and sediment was determined using the technique described by Strickland and Parsons (1972). The measurement results are presented as the mean (± standard deviation).

### Laboratory analysis

Amphioxi were kept alive in aquaria. Thus, morphological and meristic taxonomical characters were observed and recorded in live individuals, which allowed more accurate observations than individuals preserved in formaldehyde or alcohol. To record morphometric measurements, individuals were placed in a 300 mL beaker containing seawater with 12 drops (approx. 600 µL) of clove oil added as a sedative. The individuals were then transferred to a Petri dish (5–7 cm in diameter) with seawater and observed under a Nikon SMZ25 stereomicroscope. Photographs of each individual were taken and later processed with NIS-Elements imaging software for the analysis of morphometric traits.

Amphioxi were identiﬁed based on typical morphological characters, following Poss and Boschung (1996): 1) length; 2) total, preatriopore, atriopore-to-anus and postanal number of myotomes; 3) number of dorsal and preanal fin chambers; and 4) number of gonads. Based on the wide size range and the high variability of morphological characters observed in the collected specimens, we hypothesized that these might represent more than one morphotype. To explore this possibility, we examined additional characters, including 5) preatriopore, atriopore–anal distance, 6) postanal region length, 7) length of super- and sub-caudal fins, 8) body depth, 9) height of the caudal fin, 10) length and height of the rostral fin, 11) height and width of the tallest dorsal fin chamber, 12) the tallest preanal fin chamber, and 13) angle between the dorsal and super-caudal fin and between the preanal and sub-caudal fin. All counts and measurements were taken on the left side of the specimens, following Tchang and Koo (1936) and Zhang et al. (2006). Wet body weight (FW) was recorded to the nearest 0.001 g with an electronic scale (Ohaus Explorer, Florham Park, NJ, USA). Prior to weighing the specimens, excess water was removed with paper towels.

### Sex determination

The criteria proposed by Henmi and Yamaguchi (2003) were used to sex mature individuals. Immature and unmatured specimens were classified as undifferentiated.

### Statistics

The mean, standard deviation and coefficient of variation of each morphological trait were calculated separately by sex. Differences between sexes in each morphometric variable were tested with Student´s t-test (α = 0.05) (STATISTICA v 8.0). To examine the relationship between total length (TL) and weight (W) of the specimens, the power equation *TW*= a*TL^β^* was fitted to the data. In this equation, a is the average condition factor and *β* is the coefficient of allometry; a *β* value equal to 3 indicates an isometric growth pattern, whereas a value significantly different from 3 denotes allometric growth. Thus, the equation parameters and their 95% confidence intervals (CI) were estimated (Aguirre-Villaseñor et al. 2006, Esmaeili and Ebrahimi 2006), and the estimated *β* was subjected to Student’s t-test (Zar 1999) to determine whether growth was isometric or allometric. As allometric changes are related to the chronology of important life-history events and therefore reflect an ontogenetic response to functional demands, allometric analyses were conducted separately for females, males, both sexes, undifferentiated individuals, and for the entire sample.

To examine the size structure of the individuals, TL (mm) data were grouped in 2-mm class intervals separately for males and females; for undifferentiated individuals, a 1-mm class interval was used. To determine the expected number of TL groups, a multinomial probability density distribution was constructed as described by Haddon (2001). The 95% confidence interval for each group was calculated using Student’s t distribution. To test whether the sex ratio deviated significantly from 1:1, a chi-square test was carried out (α < 0.05) (Sokal and Rohlf 1995).

## Results

### Habitat

**Figure 2.**
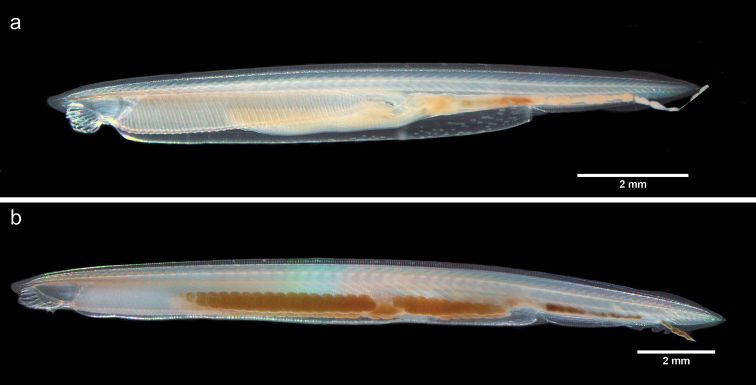
Amphioxus *Branchiostoma californiense*: **a** immature organism **b** mature organism (female).

*Branchiostoma californiense* individuals (Fig. 2) were found within and at the margins of patches of *H. wrightii*. Amphioxus abundance decreased with the distance from seagrass patches: 10 m away from a sampled patch, only a few individuals were found; 50 m or farther away from a sampled patch, no amphioxi were found in bare sediments. The average density of amphioxi in seagrass patches was 80 ind. m^-2^, for an estimated total abundance of 1920 individuals for the entire seagrass patch sampled. Temperature average in 24 hours was 23.1 ± 0.11 °C, with minimum and maximum values of 20.80 and 26.90 °C, respectively; During the survey, the average water surface temperature was 22.90 ± 0.11 °C, with minimum and maximum values of 22.04 and 24.44 °C, respectively; pH was 8.44 ± 0.39; salinity was 36.89 ± 0.31 ups; and chlorophyll *a* concentration was 0.22 ± 0.04 mg m^-3^ in seawater and 1.93 ± 0.44 mg/g in sediments.

### Amphioxus characteristics

We collected 18 male and 19 female specimens, for a 0.94:1 male-female ratio, plus 23 sexually undifferentiated individuals. The TL ranged between 15.88 and 28.44 mm (19.76 ± 3.33 SD mm) in females, 11.7–27.9 mm (19.15 ± 3.78 SD mm) in males and 12.8–20.05 (14.97 ± 2.04 mm) in undifferentiated individuals, with no significant difference between males and females (t-test = 0.52; df = 35; *p* = 0.60). The total fresh weight (TW) ranged between 0.0143 and 0.1118 g (0.04 ± 0.03 g) in females and 0.014–0.08 g (0.04 ± 0.03 g) in males, with no significant difference between their means (t-test = 0.20; df = 35; *p* = 0.84). The TW of undifferentiated individuals ranged between 0.004 and 0.05 g (0.02 ± 0.01 g).

**Figure 3.**
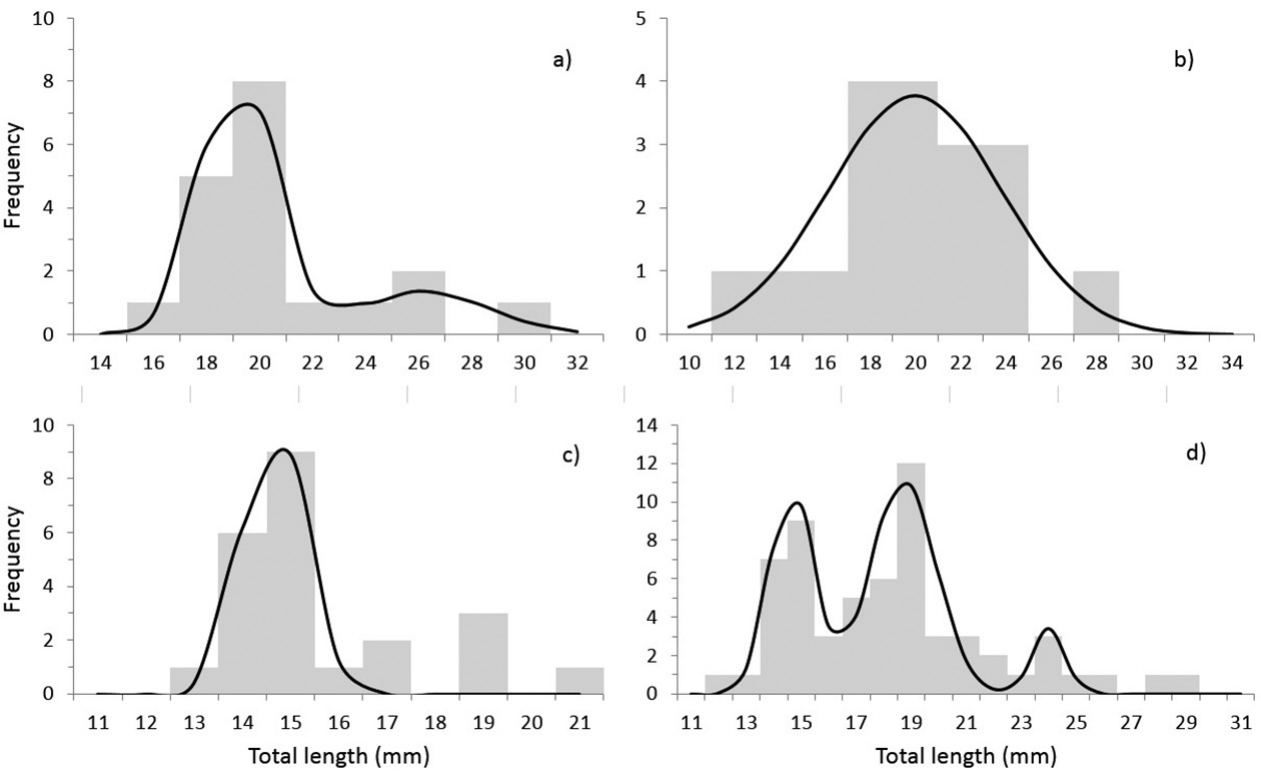
Length frequency intervals. Structure of size: **a** females **b** males **c** undifferentiated **d** all population, specimens of *Branchisotoma californiense* in Bahía Balandra, southern Baja California Peninsula.

**Figure 4.**
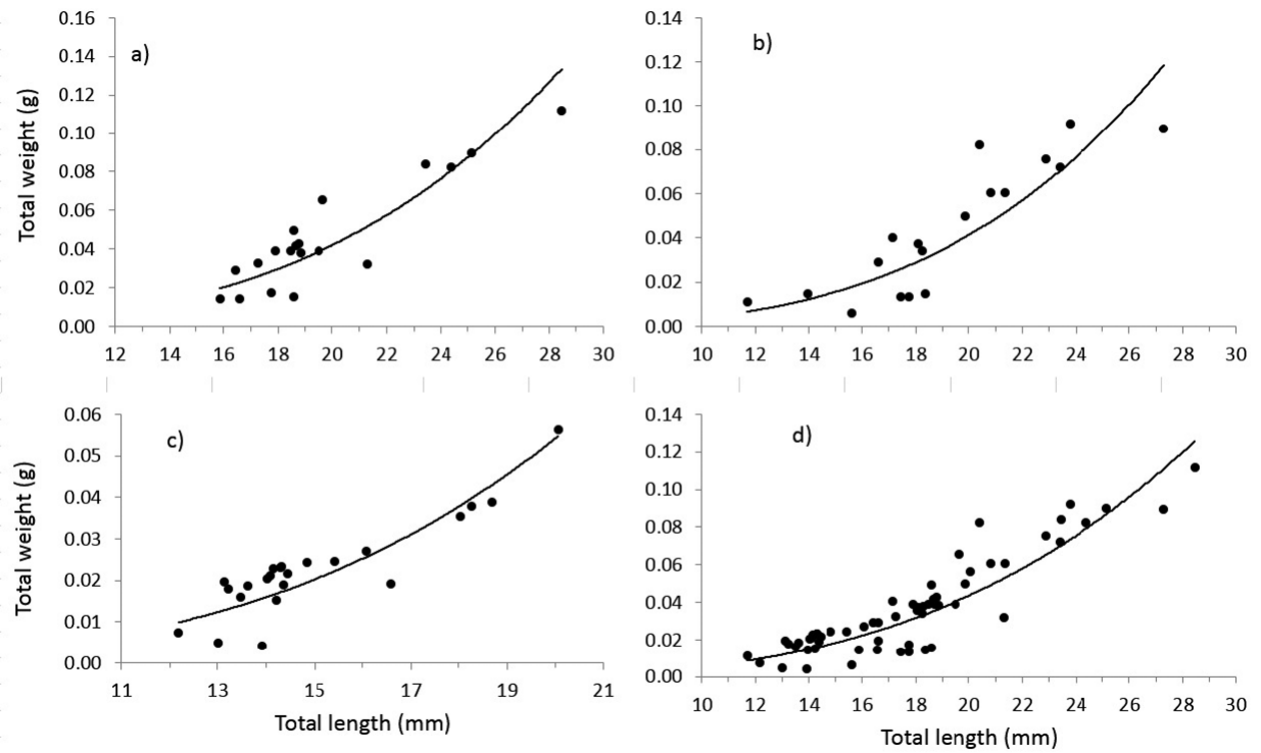
Length-weight relationship for: **a** females **b** males **c** undifferentiated and **d** all population specimens of *Branchisotoma californiense* in Bahía Balandra, southern Baja California Peninsula.

Analysis of the size structure of *B. californiense* revealed the following: 1) two size groups were identified in females, the first group with an average TL of 19.15 mm (CI = 18.50–19.81 mm, s = 2.51), and the second with an average TL of 26.11 mm (CI = 24.91–27.32 mm, s = 1.39); 2) a single size group was identified in males with an average value of 19.97 mm (CI = 18.87–21.91 mm, s = 3.81); 3) a single size group was also identified in sexually undifferentiated individuals, with an average TL of 14.63 mm (CI = 14.40–15.0 mm, s = 0.65); and 4) three size groups were identified in the overall population, with the following average TL: 14.66 mm (CI = 14.45–14.87 mm, s = 0.81), 18.73 mm (CI = 18.42–19.03 mm, s= 1.18), and 23.99 mm (CI = 23.85–24.15 mm, s = 0.59) (Fig. 3).The relationship between total length and weight (TL-W) in females (*N* = 19), males (*N* = 18), and sexually undifferentiated specimens (*N* = 23) of *B. californiense* revealed an allometric growth pattern. The slopes were statistically analyzed between both sexes and no significant differences were found (t-test, P < 0.05), between males (*β* = 3.39, SE = 0.60) and females (*β* = 3.28, SE = 0.54); consequently, the TL-W relationship can be expressed jointly (a = 1.78E-06, IC = 1.69E-07-1.88E-05; *β* = 3.35, IC = 2.56-4.15); and the TL-W relationship was equally allometric; whereas a similar analysis over the entire sample (*N* = 60) showed an isometric growth pattern (Table 1, Fig. 4).

**Table 1.** Total length-weight (TL-W) relationships for individuals of *Branchiostoma californiense* in Bahía Balandra, southern Baja California Peninsula.

Individual	*N*	r^2^	a	CI	*β*	CI	Student’s t-test	*P* value
Females	19	0.66	2.23E-6	7.49E-8–6.66E-5	3.28	2.14–4.42	44.51	< 0.007
Males	18	0.64	1.58E-6	3.79E-8–6.63E-5	3.39	2.12–4.66	56.88	< 0.005
Undifferentiated	23	0.51	1.84E-6	3.64E-8–9.30E-5	3.43	1.98–4.88	182.34	< 0.001
Whole Population	60	0.67	5.22E-6	1.09E-8–2.48E-5	3.01	2.46–3.55	4.06	< 0.07

*N* = number of data, CI = confidence interval, Student’s t-test was estimated with *df* = 1.

**Figure 5.**
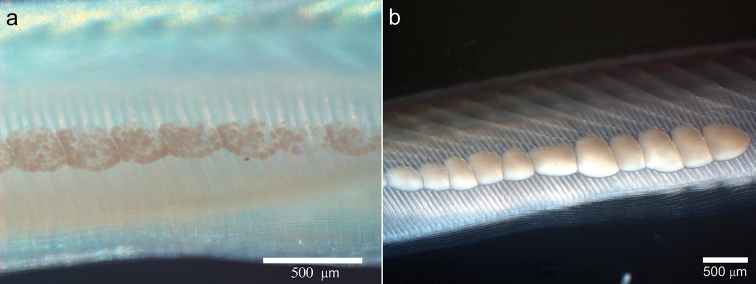
Sexual differentiation in amphioxus *B. californiense*, mature individuals: **a** female, with signs of some gonads having spawned (right side of the picture) **b** male with well-developed gonads (right side of the picture) and some gonads in the process of development (left side of the picture).

Thirty-seven individuals showed sexual dimorphism. Females had well-developed, beige-colored gonads, with oocytes visible under the stereoscope (Fig. 5). In males, gonads were whitish with no visible granules inside (Fig. 5). Individuals were bilaterally symmetrical, with well-developed gonads on both sides, although gonads were often more numerous on the right than on the left side, a trait usually associated with the maturation stage of the gonads. Spawning individuals had fewer gonads. The minimum size at sexual maturity was 11.70 mm in males and 15.88 mm in females; the largest gonad-bearing individuals measured 28.44 mm in females and 27.29 mm in males. Asynchronous gonadal maturation was observed in some individuals that bore maturing, mature and spawning gonads.

### Taxonomical characters

The myotome formula of *B. californiense* was preatriopore 44.42 (40–49) + atriopore-anal 14.57 (13–19) + postanal 8.53 (6–10) = total 67.52 (59–76). Dorsal fin of moderate height. Dorsal fin chambers 389 (345–442). Preanal fin chambers 52.98 (36–74). Body length to body depth ratio: 11.83 (8.35–18.64) (Table 2). Maximum number of gonads 37 in females, 35 in males (Tables 2, 3).

**Table 2.** Comparative data of the more relevant morphometric characteristics considered for traditional taxonomy of amphioxus for *Branchiostoma californiense*, including previous reports and those specimens caught in Bahía Balandra, southern Baja California Peninsula, Mexico. Values are given in mean (range). In bold the main differences of this study with respect to the other studies.

	Andrews (1893)	Kirkaldy (1895)	Hubbs (1922)	Poss and Boschung (1996)	Del Moral-Flores et al. (2016)	Galván-Villa et al. (2017)	This Study
TL (mm)	57–70	74 max.	37.5–83.5	No data	83.5	24.1–40.1	**11.70–28.44**
Total Myotomes	68 (64–69)	71 (69–73)	68–74	70.1(64–78)	67 (64–71)	69 (66–72)	67 (**59**–76)
Preatriopore myotomes	44 (42–45)	44–45	43–48	44.3 (42–47)	40 (40–45)	43 (39–46)	44 (40–**49)**
Myotomes between atriopore and anus	16 (13–16)	16–19	16–19	16.7 (13–19)	18 (14–19)	17 (16–19)	**14** (13–19)
Postanal myotomes	9 (8–9)	8–9	9.1 (7–11)	9 (8–9)	9 (8–9)	9 (9–10)	8 (**6**–10)
Dorsal fin chambers	No data	No data	337 (312–374)	355 (317–419)	355 (317–419)	364 (343–395)	**388 (345–442)**
Preanal fin chambers	No data	No data	50	44 (35–19)	44 (35–59)	58 (56–61)	53 (36–**74**)
Gonad pouches	No data	31	33 (273–6)	36 max.	36 max.	26 (18–31)	28 (**16–37**)
*N*	7	10	22	57	No data	5	60

**Table 3.** Meristic and non-meristic data of 37–60 individuals of *Branchiostoma californiense* found on seagrass *H. wrightii* at southern Baja California Peninsula.

Characteristics	*N*	Minimum – Maximum	Mean	SD	SE	CV
Number of myotomes anterior to atriopore	60	40–49	44.42	2.35	0.3	0.05
Number of myotomes between atriopore and anus	60	13–19	14.57	1.35	0.17	0.09
Number of myotomes posterior to anus	60	6–10	8.53	0.89	0.12	0.10
Total number of myotomes	60	59–76	67.52	3.29	0.41	0.05
Number of dorsal fin chambers	60	345–442	389	22.38	2.89	0.06
Number of preanal fin chambers	60	36–74	52.98	8.64	1.12	0.16
Number of gonads on the left side	37	16–37	28.27	4.5	1.84	15.74
Number of gonads on the right side	37	7–35	28.5	4.9	0.73	17.06
Length of body	60	11.70–28.44	17.74	3.73	0.48	0.21
Length of preatriopore region	60	8.54–20.89	12.92	2.71	0.35	0.21
Length of atriopore-anal-region	60	2.03–5.58	3.41	0.83	0.11	0.24
Length of postanal region	60	0.94–2.32	1.41	0.3	0.04	0.21
Lenght of super-caudal fin	60	1.31–3.46	2.01	0.46	0.06	0.23
Length of sub-caudal fin	60	1.46–4.18	2.43	0.5	0.06	0.21
Depth of body	60	0.75–2.22	1.52	0.3	0.04	0.20
Lenght of rostral fin	60	0.18–0.52	0.36	0.08	0.01	0.23
Height of rostral fin	60	0.11–0.44	0.24	0.08	0.01	0.33
Height of caudal fin	60	0.69–1.57	0.99	0.21	0.03	0.21
Length to depth of body	60	8.35–18.64	11.83	1.85	0.23	15.67
Heigth to width of tallest dorsal fin chamber	60	0.50–6.33	3.17	1.09	0.14	34.44
Height to width of tallest preanal fin chamber	60	1.0–4.5	2.39	0.72	0.09	30.02
Angle between dorsal and super-caudal fins	60	150.37–180.52	168.97	37.74	0.79	0.04
Angle between preanal and sub-caudal fins	60	111.19–184.31	167.68	9.09	1.17	0.05

### Morphological characters

**Figure 6.**
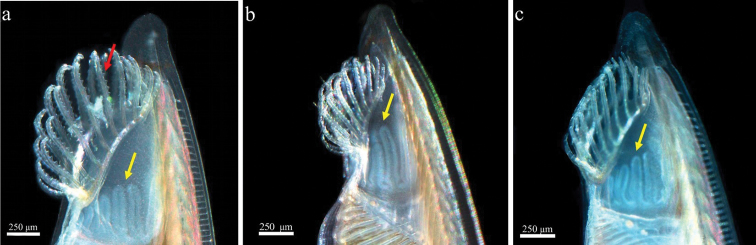
Morphology oral region. Serrations of the cirri (red arrow) (**a**). Velar tentacle (yellow arrow) of the wheel organ having a singular variation in the shape, some projections were different in length and shape (**b, c**).

**Figure 7.**
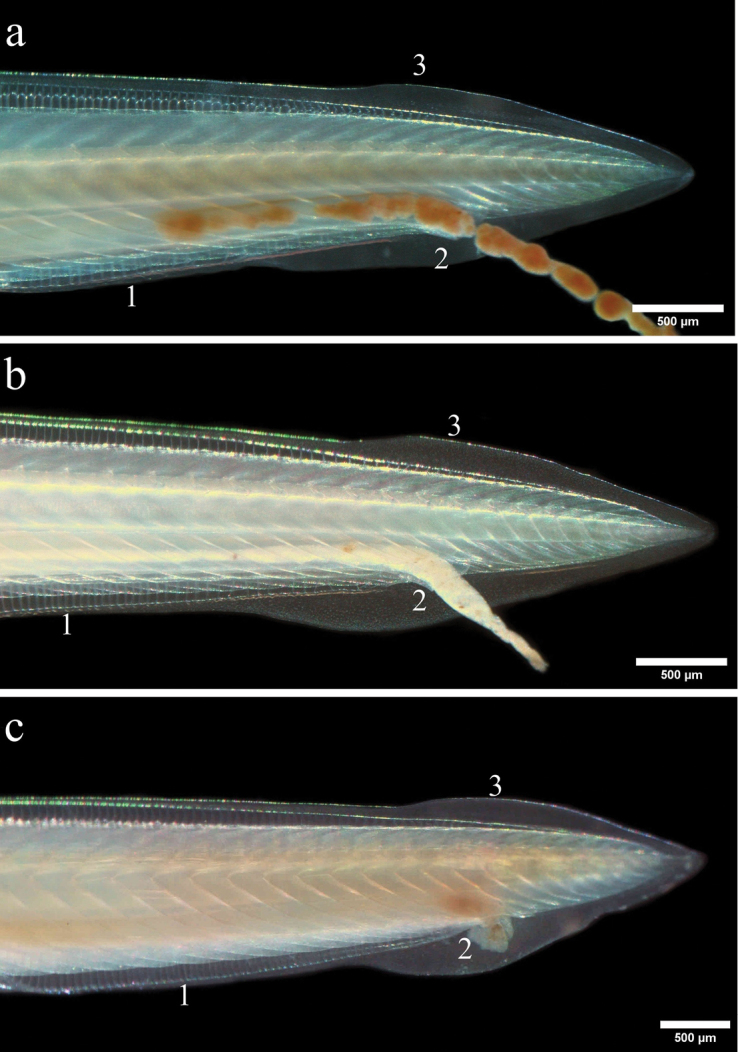
Morphology caudal region (**a–c**). Posterior part of *B. californiense* showing differences in caudal fin and preanal fin chambers: (**1**) preanal fin chambers, (**2**) anus, (**3**) lobe dorsal fin variability.

*Branchiostoma californiense* has an elongated body. The notochord extends beyond the oral hood and forms a well-developed rostral process. Buccal region with numerous fine cirri (up to 24), most of them longer than the tip of the rostrum (Fig. 6). Cirri with (75% of the individuals) or without (25%) serrations (Fig. 6). Rostral fin thickened, with a round end, although some variations (i.e., less thickened and larger rostral fins) were observed. Velar tentacle of the wheel organ with variable shape, some projections with varying length and shape (Fig. 6). Caudal fin with long, shallow expansions of dorsal and ventral fins. Anus well posterior to the center of the ventral lobe of the caudal fin (Fig. 7). Body color fully translucent. No significant differences between males and females in the morphometric variables examined were found (see Suppl. material 1).

## Discussion

Since 1932, *B. californiense* has been reported from 68 localities in the Baja California Peninsula, 31 of those in the Gulf of California and 37 along the Pacific coast (Poss and Boschung 1996, GBIF 2018), but no study had previously recorded the occurrence of amphioxus in a seagrass habitat. In addition, our study is one of the very few providing biological and ecological information, and it documents the most recent record of this species in the Gulf of California. Habitats more commonly reported for amphioxus are shallow subtidal sand flats in tropical, subtropical and temperate waters. Amphioxi usually prefer coarse sand with fairly fast water flow, although they can also be found in silty sediments (Berrill 1987, Nishikawa et al. 1997, Chen 2007). Most species have benthic habits, and the adults live burrowed in the sand, gravel, or shell deposits (Vargas and Dean 2010, Desdevises et al. 2011). *Branchiostoma californiense* is most commonly found in sandy flat bottoms (Vargas and Dean 2010, Galván-Villa et al. 2017). In contrast, all the specimens in this study were collected in patches of the seagrass *Halodule wrightii*; this reveals the wider habitat range and tolerance to seagrass habitat conditions of the amphioxus *B. californiense*. Remarkably, no individuals were found in bare areas.

Variables recorded during our sampling outline the environmental conditions in the seagrass patches where the population of *B. californiense* lives in Bahía Balandra. The temperature during the sampling period corresponds to the transition period (May-June) between the cold (December–April) and warm (July-November) seasons in the Gulf of California (Martínez-López et al. 2001). The concentration of chlorophyll *a* in water was low, as was already reported for the same dates (Martínez-López et al. 2001), but the concentration in sediment inside the patch was high. These values denote the productivity level prevailing at the time of sampling in this habitat occupied by amphioxus.

It is known that seagrass meadows are one of the most productive marine habitats (Larkum et al. 2006), and they are thought to play a key role in maintaining populations of commercially exploited marine fishes and invertebrates by providing crucial ecosystem services, including: (1) permanent habitat allowing completion of the life cycle, (2) nursery areas for the development of juvenile stages, (3) feeding grounds for various life-history stages and (4) refuge from predators (Jackson et al. 2001). In addition, the seagrass canopy structure attenuates hydrodynamic forces, and its below-ground structures stabilize the sea floor, creating milder growing conditions (Brun et al. 2009). As active filter feeders, amphioxi living in the seagrass canopy benefit from the local reduction in the flow regime (i.e., higher sediment stability) and the lower disturbance that allow them to obtain food by filtering suspended particles from the surrounding waters. Additionally, amphioxi likely find refuge and suitable habitat inside and at the edge of seagrass patches, as has been observed in other filter-feeder organisms (Irlandi and Peterson 1991, Irlandi 1996, Brun et al. 2009, González-Ortiz et al. 2014, Jiménez-Ramos et al. 2018).

There is high variability in the size structure of *B. californiense* populations across its distribution range. The largest sizes (83.5 mm) have been recorded in northern latitudes (Monterey Bay, California) (Hubbs 1922), whereas the smallest sizes (5 mm) were reported by Vargas and Dean (2010) in southern latitudes (Costa Rica). Galván-Villa et al. (2017) reported sizes ranging from 24.1 to 40.1 mm at a depth of 5–8 m in the central Mexican Pacific. Our specimens are the smallest (11.70 to 28.44 mm) reported for the Gulf of California and the Mexican Pacific; they may represent recent recruits, as Vargas and Dean (2010) found for Costa Rica. The smallest sexually mature specimen in our sample was an 11.70 mm (TL) male. Thus, it can be assumed that juveniles are likely smaller than 11 mm. The lifespan for *B. californiense* is unknown, but according to growth models of *B. belcheri* (Chen et al. 2008), the body size range of one year and, two and three-years-old individuals was 5–28 mm, 28–38 mm and 38–45 mm length, respectively. Therefore, it is likely that *B. californiense* individuals in our study area (11.70–28.44 length) probably belong to a one-year-old group compared, for example, to the report of Hubbs (1922) (37.5–83.5 length), which individuals belong to the two- or three-years-old group. However, it is also possible that body size differences can be related to latitudinal variation following the Bergman´s rule, bigger individuals in higher latitudes (Lomolino et al. 2006). Thus, northern individuals (36°N; Hubbs 1922) were larger in comparison to the lower latitude at our study site in the Gulf of California (24°N).

This study documents, for the first time, the TL-W relationship in a natural population of *B. californiense*. This information is useful for understanding the relationship and expected trends of length and weight under natural conditions and helps to elucidate whether the species exhibits an allometric or isometric growth pattern. A positive allometric growth pattern (*β* > 3) means that the organism grows in weight proportionately faster than it does in length (NOAA 2006); this growth pattern was observed in the females, males and undifferentiated individuals in our sample. An isometric growth pattern (*β* = 3) means that any increment in length is associated with a proportional increment in weight (NOAA 2006); this growth pattern was observed when we analyzed the entire sample of *B. californiense*. The change from allometric (males, females and undifferentiated, or both sexes jointed) to isometric (all population specimens) may be associated to the different TL intervals among groups; the organisms undifferentiated had a range of TL (12–20 mm) 50% smaller than for males and females (12–28 mm and 16–29 mm, respectively). When the different groups were independently analyzed the variability in TL-W data influenced an apparent allometric condition. However, the analysis based on all population specimens allowed the estimate of an isometric growth pattern, which was influenced by a TL interval wider. Several factors, such as age, body shape, amount of body fat, sex, maturity stage, season, and abiotic variables (e.g., temperature, salinity and available nutrient), influence the value of *β* in the total length-total weight relationship, causing seasonal and between-habitat variations (Froese 2006, Froese et al. 2014, He et al. 2008).

Analysis of the size structure of the *B. californiense* sample revealed different groups, which might correspond to different cohorts being present in the population, as has been suggested for fish populations (Chang et al. 2012, Le Bris et al. 2015). In this study, three distinct length groups were identified, suggesting the presence of three distinct cohorts (two cohorts for females and one for males). A single size group was identified in the undifferentiated specimens, with an average length (14.63 ± 2.04 SD mm) smaller than that of females (19.76 ± 3.32 SD mm) or males (19.15 ± 3.77 SD mm). These smaller individuals might constitute the new recruits in the population; this hypothesis could have been verified had field data on age and reproduction been also obtained during the sampling. The fact that 62% of the specimens in our sample were sexually mature adults and that the size structure shows the presence of different cohorts in the population indicates that part of the cycle life of *B. californiense* takes place in this habitat. Thus, based on our results, we propose that the patches of *H. wrightii* in Bahía Balandra provide breeding grounds for *B. californiense*.

## Diagnosis

Data obtained in this study on the meristic and morphometric characters of amphioxus significantly contribute to the knowledge of this species and will support future studies on other populations in the Gulf of California and Pacific coasts. Our data on the meristic characters of *B. californiense* show some differences from previous reports. All the characters necessary to determine the genus and species (Gill 1895, Hubbs 1922, Galván-Villa et al. 2017) could be observed in all our specimens. However, we recorded a wider range of total and postanal myotomes. These differences may stem from methodological issues rather than being due to actual differences in the organisms, as other authors reported meristic and morphometric data as recorded in preserved individuals (Poss and Boschung 1996, Galván-Villa et al. 2017). Preservation makes observation, counting, and measurement more difficult as the specimens turn whitish and opaque upon preservation, and tissues shrink because of fixation.

Poss and Boschung (1996) observed high morphometric variation between specimens of *B. californiense* without a well-defined geographical pattern. In our study, we also found high variation between individuals in morphometric characters, especially in the rostral and caudal fins (angles), body depth and preanal fin chambers; such variations suggest the presence of different morphotypes. For example, those characters have been considered diagnostic for distinguishing between the two species *B. japonicum* Willey, 1897 and *B. belcheri* Gray, 1847 (Zhang et al. 2006). On the other hand, Galván-Villa et al. (2017) observed an increasing north-south trend in the number of dorsal and preanal fin chambers of specimens collected in Mexico. However, our observations were performed on live organisms, and we found the largest numbers of dorsal (345–442) and preanal (36–74) fin chambers recorded so far, whereas Galván-Villa et al. (2017) reported smaller numbers of both dorsal and preanal fin chambers in an area much farther south.

Amphioxus is usually a bisexual or gonochoristic organism; the mature gonad is whitish in males and yellow in females (Zhang et al. 2001), as confirmed in our study. We also found a higher variation in the number of gonads than previously reported, thus widening the range previously reported to 16–37. A range of 18–31 gonads was reported by Galván-Villa et al. (2017) and a range of 27–36 gonads by Hubbs (1922). This difference may be related to either the maturation stage or the manner in which observations were made. In our study, we observed asynchronous gonadal development in some specimens, which showed gonadal remnants (still bearing a few reproductive cells, indicating that the organism had already spawned), mature and maturing gonads. In our study, we took all the gonadal development stages into account when counting the number of gonads.

The velar tentacles of the wheel organ showed some variations in the length and shape of the projections. Because the velar tentacles are retractable structures, such variations could well be due to the specimens showing different degrees of relaxation in response to the clove oil used to sedate them. By contrast, when observations are made on fixed organisms the velar tentacles will always be contracted. The filtering system of amphioxus consists of a mouth surrounded by a circular velum with velar tentacles (oral cirri), the branchial basket (pharynx) and the atrium chamber (Orton 1913, Weel 1937, Chen 2007). We found fewer oral cirri (up to 24) than the number reported by Galván-Villa et al. (2017) (up to 40). Hubbs (1922) described the oral hood and cirri becoming smaller with age, but we did not observe such trends in our study. Finally, 25% of the specimens in our sample did not show serrations in the cirri, and some showed damage to the caudal fin. Chen (2007) indicated that an increased content of suspended solids can cause physical damage to filter-feeding structures, such as the absence of serrations in the cirri of amphioxus, which coincides with our observations.

## Conclusions

The occurrence of the amphioxus *B. californiense* in the Gulf of California was first reported 86 years ago. Our study is the first to provide information on its biology, particularly the population’s size structure. Our results show that *B. californiense* is not strictly associated with bare sandy areas, as had been previously reported for the Gulf of California, but that seagrass beds are also an important habitat offering distinct structural characteristics for amphioxus. Further efforts of sampling both in time and space are needed to study the relationship between *B. californiense* and seagrasses to elucidate its ecological relevance and the role of *B. californiense* as active filter feeders in this ecosystem.

## Supplementary materials

Supplementary material 1 Comparative morphometric variables of *Branchiostoma californiense* Data type: measurement Explanation note: Differences between sex for each morphometric variable (t-Test). https://binary.pensoft.net/file/328982This dataset is made available under the Open Database License (http://opendatacommons.org/licenses/odbl/1.0/). The Open Database License (ODbL) is a license agreement intended to allow users to freely share, modify, and use this Dataset while maintaining this same freedom for others, provided that the original source and author(s) are credited.Lucía Campos-Dávila, Claudia J. Pérez-Estrada, Ricardo Rodríguez-Estrella, Enrique Morales-Bojórquez, Fernando G. Brun-Murillo, Eduardo F. Balart
