# Supplementary material for: Seagrass Halodule wrightii as a new habitat for the amphioxus Branchiostoma californiense (Cephalochordata, Branchiostomidae) in the southern Gulf of California, Mexico
Source: Zookeys. 2019 Aug 29;873:113–31. doi: 10.3897/zookeys.873.33901 (PMC6728395; doi:10.3897/zookeys.873.33901)
Supplement: Supplementary material 1 [file zookeys-873-113-s001.docx]

Supplementary 1. Comparative morphometric variable of *Branchiostoma californiense*. Differences between sex for each morphometric variable (t-Test).

| **Characteristics** | Female | | Male | |  |  |  |
| --- | --- | --- | --- | --- | --- | --- | --- |
|  | Mean | S.D. | Mean | S.D. | t-value | df | *p* Value |
| Length of body | 19.76 | 3.33 | 19.15 | 3.78 | 0.53 | 35 | 0.60 |
| Weight of body (gr) | 0.05 | 0.03 | 0.04 | 0.03 | 0.20 | 35 | 0.84 |
| Length of preatriopore region | 14.33 | 2.42 | 14.00 | 2.74 | 0.40 | 35 | 0.69 |
| Length of atriopore-anal-region | 3.92 | 0.75 | 3.62 | 0.89 | 1.11 | 35 | 0.27 |
| Length of postanal region | 1.51 | 0.27 | 1.53 | 0.31 | **-**0.23 | 35 | 0.82 |
| Depth of body mm | 1.68 | 0.27 | 1.60 | 0.27 | 0.90 | 35 | 0.37 |
| Lenght of super-caudal fin | 2.19 | 0.37 | 2.24 | 0.51 | **-**0.38 | 35 | 0.71 |
| Length of sub-caudal fin | 2.66 | 0.43 | 2.61 | 0.56 | 0.27 | 35 | 0.79 |
| Wide Caudal fin | 1.10 | 0.18 | 1.08 | 0.21 | 0.24 | 35 | 0.81 |
| Angle between dorsal and super-caudal fins | 170.86 | 4.05 | 168.77 | 7.76 | 1.04 | 35 | 0.31 |
| Angle between preanal and sub-caudal fins | 166.47 | 4.92 | 167.75 | 5.03 | **-**0.78 | 35 | 0.44 |
| Notocord-Point | 0.12 | 0.06 | 0.11 | 0.03 | 0.85 | 35 | 0.40 |
| Lenght of rostral fin | 0.36 | 0.06 | 0.37 | 0.10 | **-**0.48 | 35 | 0.63 |
| Height of rostral fin | 0.26 | 0.08 | 0.27 | 0.08 | **-**0.30 | 35 | 0.77 |
| Number of myotomes anterior to atriopore | 44.05 | 1.68 | 44.72 | 2.56 | **-**0.94 | 35 | 0.35 |
| Number of myotomes between atriopore and anus | 15.05 | 1.68 | 14.39 | 1.04 | 1.43 | 35 | 0.16 |
| Number of myotomes posterior to anus | 8.26 | 0.73 | 8.72 | 1.07 | **-**1.53 | 35 | 0.14 |
| Number of dorsal fin-chambers | 382.79 | 19.71 | 391.22 | 24.77 | **-**1.15 | 35 | 0.26 |
| Number of preanal fin-chambers | 56.53 | 7.53 | 53.72 | 9.81 | 0.98 | 35 | 0.33 |
| Width of tallest dorsal fin-chamber | 0.06 | 0.04 | 0.05 | 0.01 | 1.29 | 35 | 0.20 |
| Height of tallest dorsal fin-chamber | 0.14 | 0.03 | 0.14 | 0.03 | 0.00 | 35 | 1.00 |
| Width of tallest ventral fin-chamber | 0.07 | 0.01 | 0.06 | 0.02 | 0.41 | 35 | 0.68 |
| Height of tallest ventral fin-chamber | 0.16 | 0.05 | 0.16 | 0.04 | 0.63 | 35 | 0.54 |
